# Complete Genome Sequences of Two Crimean-Congo Hemorrhagic Fever Viruses Isolated in China

**DOI:** 10.1128/genomeA.00571-13

**Published:** 2013-08-01

**Authors:** Zhaorui Zhou, Weiwei Meng, Fei Deng, Han Xia, Tianxian Li, Surong Sun, Manli Wang, Hualin Wang, Yujiang Zhang, Zhihong Hu

**Affiliations:** State Key Laboratory of Virology and China Centre for General Viruses Culture Collection, Chinese Academy of Sciences, Wuhan, Chinaa; Center for Disease Control and Prevention of Xinjiang Uygur Autonomous Region, Urumuqi, Xinjiang, Chinab; Xinjiang Key Laboratory of Biological Resources and Genetic Engineering, College of Life Science and Technology, Xinjiang University, Urumuqi, Xinjiang, Chinac

## Abstract

Here, we report the complete genome sequences of two Crimean-Congo hemorrhagic fever virus (CCHFV) strains, 79121M18 and YL04057, isolated in Xinjiang, China. Sequence analysis showed that they represent a genotype of CCHFV that has not been reported before.

## GENOME ANNOUNCEMENT

Crimean-Congo hemorrhagic fever virus (CCHFV) is a tick-borne pathogen, a member of the *Nairovirus* genus in the *Bunyaviridae* family, possessing a tripartite RNA genome of L, M, and S segments. The virus was first discovered in the Crimean peninsula in 1944 and has been reported in >30 countries (1). According to S segment phylogeny, all CCHFVs can be grouped into seven groups: Asia 1 and 2, Africa 1, 2, and 3, and Europe 1 and 2 (2, 3). In China, the first CCHF case was reported in 1965 in Xinjiang Province and then sporadic outbreaks occurred in that area. So far, only one full-genome sequence of a Chinese CCHFV isolate (C-68031) has been reported (4).

Here, we report the full-genome sequences of two new isolates, 79121M18 and YL04057, both from Xinjiang, China. Strain 79121M18 was isolated from a rodent (*Euchoreutes naso*) in 1979, and YL04057 was isolated from ticks (*Hyalomma asiaticum*) in 2004. The viral RNA was isolated as described previously (5), and cDNA was obtained with random primers. To determine the full genome sequence of the isolates, a total of 23 pairs of primers (3 for S, 8 for M, and 12 for the L segments) were designed based on the alignment of CCHFV full-genome sequences in GenBank with overlapping for every adjacent fragment. The PCR products were purified and cloned into the pGEM-T Easy vector (Promega) and sent for sequencing. DNAStar (version 7.10), BioEdit (version 7.053), and MEGA 5.10 were applied for the genomic sequence analysis. As the sequences of the untranslated regions (UTRs) of some reference isolates are not available, the coding region of each segment was used for genome comparison analysis.

The genomes of 79121M18 and YL04057 are almost identical, with lengths (in nucleotides [nt]) (and identities) of 121,561 (98.9%), 5,377 (99%), and 1,673 (97.9%) for the L, M, and S segments, respectively. Phylogenetic analysis based on the S segment sequences showed that 79121M18 and YL04057 cluster together with other Chinese isolates and neighboring countries’ isolates in the group of Asia 2, with nt identities ranging from 93% to 99.5%; also, they were closer to isolates of Asia 1 (with nt identities of 88.8% to 91.7%) than to those of the European and African groups (identities of 83.8% to 88.9%). For M segment sequences, they share high nt identities of 99.8% (79121M18) and 98.9% (YL04057) with the Chinese isolate 7001 and are closer to African strains, including Semunya (Ugandan isolate) and ArD8194 and ArD15786 (Senegalese isolates), with nt identities ranging from 80.6% to 81.9%; however, they are distinguished from all other groups with low identity (<71.2%). For L segment sequences, 79121M18 and YL04057 form a distinct group with 98.9% nt identity and are distinguished from all other isolates, with nt identities ranging from 78.2% to 88.8%.

In summary, 79121M18 and YL04057 are two closely related CCHFV isolates. They are clustered into the Asia 2 group based on phylogeny analysis of the S segment, but the distinctive sequences of the M and L segments indicate that they represent a new genotype of CCHFV.

### Nucleotide sequence accession numbers. 

The complete virus genome sequences have been submitted to GenBank under accession no. GU477492 to GU477494 for strain 79121M18 and FJ562093 to FJ562094 for strain YL04057.
